# SARS-CoV-2-Specific Antibody Prevalence and Symptoms in a Local Austrian Population

**DOI:** 10.3389/fmed.2021.632942

**Published:** 2021-05-24

**Authors:** Dennis Ladage, Yana Höglinger, Dorothee Ladage, Christoph Adler, Israfil Yalcin, Oliver Harzer, Ralf J. Braun

**Affiliations:** ^1^Department of Internal Medicine, Danube Private University, Krems/Donau, Austria; ^2^Heart Center, University of Cologne, Cologne, Germany; ^3^Department of Pneumology, Kliniken Maria Hilf, Mönchengladbach, Germany; ^4^Research Division for Neurodegenerative Diseases, Danube Private University, Krems/Donau, Austria; ^5^Fire Department, City of Cologne, Institute for Security Science and Rescue Technology, Cologne, Germany; ^6^Center of Biosciences, Danube Private University, Krems/Donau, Austria; ^7^Bioscientia, Institute of Medical Diagnostics, Ingelheim, Germany

**Keywords:** SARS-CoV-2, COVID-19, immunology & infectious diseases, antibody prevalence, disease symptom assessment

## Abstract

**Background:** Since December 2019 the novel coronavirus (SARS-CoV-2) is the center of global attention due to its rapid transmission and toll on health care systems and global economy. Population-based serosurveys measuring antibodies for SARS-CoV-2 provide one method for estimating previous infection rates including the symptom-free courses of the disease and monitoring the progression of the epidemic.

**Methods:** In June 2020 we succeeded in testing almost half of the population of an Austrian township (1,359 inhabitants) with a reported higher incidence for COVID-19 infections (17 PCR positive cases have been officially reported until the date of sample collection, i.e., 1.2% of the total population). We determined the prevalence of SARS-CoV-2-specific antibodies in this population, factors affecting, and symptoms correlated with prior infection. Antibodies were determined using a CE-certified quality-controlled ELISA test for SARS-CoV-2-specific IgG and IgA antibodies.

**Results:** We found a high prevalence of 9% positive antibodies among the town population in comparison to 6% of the neighboring villages. This was considerably higher than the officially known RT-PCR-approved COVID-19 cases (1.2%) in the town population. Twenty percent of SARS-CoV-2-antibody positive cases declared being asymptomatic in a questionnaire. On the other hand, we identified six single major symptoms, including anosmia/ageusia, weight loss, anorexia, general debility, dyspnea, and fever, and especially their combination to be of high prognostic value for predicting SARS-CoV-2 infection in a patient.

**Conclusions:** This population study demonstrated a high prevalence of antibodies to SARS-CoV-2 as a marker of past infections in an Austrian township. Several symptoms revealed a diagnostic value especially in combination.

The world is still in the midst of the severe acute respiratory syndrome coronavirus 2 (SARS-CoV-2) infection pandemic, with Austrian towns, such as Ischgl, acting as local epicenters. In June 2020, we succeeded in testing approximately half of the population (47%) of an Austrian township with a reported high incidence of coronavirus disease (COVID-19) infections. We determined the prevalence of SARS-CoV-2 infections in this population, factors affecting it, and the symptoms associated with prior infection. The study's design and execution were in accordance with the local ethics committee and were approved by the local and national authorities.

The township of Weißenkirchen/Wachau (1,359 inhabitants) comprises the town Weißenkirchen (926) and the communities Wösendorf (296), Joching (150), and St. Michael (23). Participants were recruited with a public call that was supported by local authorities as well as the Austrian red cross. A group of 835 participants comprising people of all ages (ranging from 7 to 89 years) with a uniform distribution of sex (48% male) was tested for SARS-CoV-2-specific immunoglobin G (IgG) and immunoglobulin A (IgA) antibodies. The participants completed a questionnaire on personal data as well as disease symptoms, their onset, and duration.

Blood samples from the study group were tested in a certified diagnostic laboratory (Bioscientia, Ingelheim, Germany) using an EC-certified semiquantitative enzyme-linked immunosorbent assay (ELISA) (Euroimmun Anti-SARS-CoV-2-ELISA IgG and IgA). Although, the reference method for screening and diagnosis of acute COVID-19 infections is reverse transcription polymerase chain reaction (RT-PCR), the detection of antibodies against SARS-CoV-2 (IgG, IgA) plays a complementary role. It is particularly important for providing epidemiological information about previous infections, especially in the early times of the pandemic, when information about the dark figure, the number of unreported cases was an unknown factor (1). Seroprevalence has been observed in patients with COVID-19 confirmed by RT-PCR, as recently reviewed (2). So far, only a few studies have assessed seroprevalence in primarily asymptomatic individuals. The numbers during the early phase of the pandemic were overall low (1.6%) even among high-risk groups of healthcare workers having frequent contact with patients with COVID-19 (3). Additionally, only up to 5% seroprevalence was discovered in smaller studies in the general population (4).

Using the sensitive and reliable laboratory-based ELISA assay, 8.5% (71/835) and 9.0% (75/835) of the participants tested in our study showed SARS-CoV-2-specific IgG and IgA antibodies, respectively (Figure 1). Both classes of antibodies were found in 5.7% (48/835) of the participants. The high number of participants with SARS-CoV-2-specific IgA antibodies could be a hint of more recent infections (5). Due to their stickiness the detection of IgA antibodies is inherently less reliable than that of IgG. Thus, these data must be treated with caution. The day of sample collection was clearly after the first pandemic peak in Austria with very low infection rates at that time. Furthermore, we excluded cases with acute disease symptoms from our study. Therefore, no acute symptomatic COVID-19 cases should be included. Consequently, we considered only participants with SARS-CoV-2-specific IgG antibodies as cases having previous contact with SARS-CoV-2.

**Figure 1:**
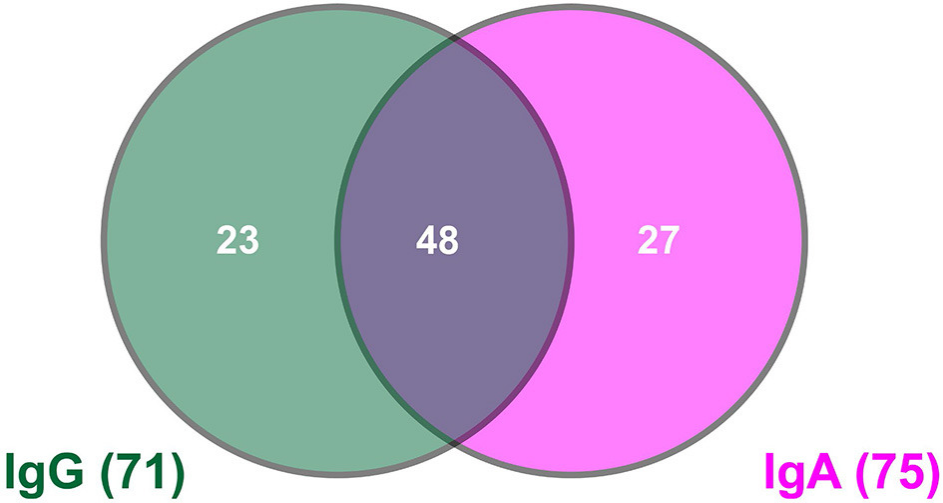
Venn diagram showing the number of cases with SARS-CoV-2-specific antibodies.

Individuals who showed SARS-CoV-2-specific IgG antibodies stated significantly more often that they either stayed abroad or in the Austrian state of Tyrol (42%, 30/71) as compared to the total tested population (26%, 206/806). Notably, the national hot-spot, Tyrol, was not the source of the virus, but other countries, mainly Israel, were sources. From those who visited Israel in early 2020, 53% (10/19) developed SARS-CoV-2-specific IgG antibodies. Thus, the virus most likely was introduced from external hot-spots into the local population, where it proliferated.

Nine percent (61/695) of the tested individuals in the township of Weißenkirchen developed SARS-CoV-2-specific IgG antibodies in contrast to 6% (10/167) in the control group of tested individuals from neighboring municipalities. Within the township of Weißenkirchen, 10% (45/458) of the participants from the town of Weißenkirchen, 38% (6/16) from St. Michael, and only 5% (6/114) from Wösendorf and 6% (4/71) of Joching showed virus-specific antibodies. Thus, as expected, the township of Weißenkirchen was more affected by COVID-19 than were the neighboring municipalities. Moreover, within the township, the infection rates could be mainly localized to the town of Weißenkirchen and the community of St. Michael. The official number of known RT-PCR-approved COVID-19 cases in the town population was 1.2% (17/1359) until the time point of sample collection. Thus, the dark figure of unknown infections in Weißenkirchen can be estimated to 7%.

Fifty-four percent (38/71) of participants with SARS-CoV-2-specific IgG antibodies were male, as compared to 48% (404/834) in the total tested population. From our data, a higher vulnerability of the male population, as has been indicated by some studies, is not evident. However, these studies were based on recent epidemiological data from Asia and an especially large population analysis in China (6). Similarly, we could not find significant influences of age, body mass index, and alcohol intake on the level of infection within the tested population.

Smokers turned out to be underrepresented among the participants with SARS-CoV-2-specific antibodies. Eight percent of participants with SARS-CoV-2-specific IgG antibodies identified themselves as smokers as compared to the 17% in the total population. Since this observation did not reach statistical significance, it remains unclear whether smoking may reduce the risk of SARS-CoV-2 infection. The current data suggest that smokers are more vulnerable and that smoking is a predictor of negative outcomes, but not necessarily for a higher susceptibility to (asymptomatic) infection (7).

Twenty percent (14/71) of participants who developed SARS-CoV-2-specific IgG antibodies self- declared not to have noticed any of the 19 different disease symptoms listed in the questionnaire (Table 1) whereas 80% (57/71) self-declared one or more disease symptoms. Although some of these symptoms may have been related to other diseases during the evaluation period, our data suggest that asymptomatic SARS-CoV-2 infections are rather uncharacteristic for the tested population. In fact, participants with SARS-CoV-2-specific IgG antibodies self-declared to have significantly more disease symptoms during the evaluation period than the total population tested in our study (Table 1). Furthermore, participants having contact with SARS-CoV-2 demonstrated anosmia/ageusia, weight loss, anorexia, general debility, dyspnea, and fever more significantly than the total tested population. The enrichment of disease symptoms becomes more distinct when comparing participants with SARS-CoV-2-specific IgG antibodies with participants lacking both virus-specific IgG and IgA antibodies (Table 1). Participants of the IgG and IgA negative group had most likely no contact to the virus before. More and larger samples will be required to confirm the prognostic values of symptoms found in other local studies (8).

**Table 1:** Disease symptoms in the tested population.

**Disease symptoms in the evaluation period (January to June 2020)**	**Number of total cases with disease symptoms**	**Number of cases lacking both SARS-CoV-2-specific IgG- and IgA antibodies but describing disease symptoms**	**Number of cases with SARS-CoV-2-specific IgG antibodies and with disease symptoms**
Cases with symptoms	532 (63.7%)	456 (61.9%)	57 (80.3%)
Cases without any symptoms	296 (35.4%)	274 (37.2%)	14 (19.7%)
Cases without data	7 (0.8%)	7 (1.0%)	0 (0.0%)
^*^Anosmia/ageusia	63 (7.5%)	35 (4.8%)	26 (36.6%)
^*^Weight loss	33 (4.0%)	21 (2.9%)	11 (15.5%)
Apathy	9 (1.1%)	5 (0.7%)	3 (4.2%)
^*^Anorexia	49 (5.9%)	33 (4.5%)	15 (21.1%)
Pneumonia	4 (0.5%)	3 (0.4%)	1 (1.4%)
^*^General debility	147 (17.6%)	119 (16.2%)	24 (33.8%)
^*^Dyspnea	51 (6.1%)	43 (5.8%)	8 (11.3%)
^*^Fever	133 (15.9%)	109 (14.8%)	20 (28.2%)
Diarrhea	105 (12.6%)	88 (11.9%)	14 (19.7%)
Stomach ache	60 (7.2%)	51 (6.9%)	7 (9.9%)
Headache / Pain in the limbs	224 (26.8%)	190 (25.8%)	26 (36.6%)
Eczema	21 (2.5%)	18 (2.4%)	2 (2.8%)
Tussis	278 (33.3%)	247 (33.5%)	25 (35.2%)
Rhinitis	301 (36.0%)	261 (35.4%)	27 (38.0%)
Somnolence	12 (1.4%)	11 (1.5%)	1 (1.4%)
Sore throat	222 (26.6%)	200 (27.1%)	16 (22.5%)
Swelling of the lymph node	45 (5.4%)	41 (5.6%)	3 (4.2%)
Nausea/vomiting	49 (5.9%)	44 (6.0%)	3 (4.2%)
Conjunctivitis	28 (3.4%)	24 (3.3%)	1 (1.4%)

*Data are based on self-declarations of tested cases. Disease symptoms are ordered according to their enrichment in cases with SARS-CoV-2-specific IgG antibodies as compared to total cases (*

* *: significant enrichments)*.

In this community-based SARS-CoV-2 population study in Austria, we found a higher seroprevalence (9%) in the town population than in the neighboring villages (6%). The seroprevalence exceeded the number of officially documented COVID-19 cases (1.2%). Considering this large sample comprising approximately half of the town population, we identified six single major symptoms, especially in combination, to be of a high prognostic value for predicting SARS-CoV-2 infection in a patient. This study has limitations; selection bias cannot be ruled out due to the voluntary nature of the study. Therefore, the estimated prevalence may be biased due to non-response or because previously symptomatic persons may have been more likely to participate. Ongoing additional population tests and follow-up tests investigating the prevalence in the town will provide further insights into the still developing and currently dynamic pandemic situation.

## Data Availability Statement

The raw data supporting the conclusions of this article will be made available by the authors, without undue reservation.

## Ethics Statement

Ethical review and approval was not required for the study on human participants in accordance with the local legislation and institutional requirements. Written informed consent to participate in this study was provided by the participants' legal guardian/next of kin.

## Author Contributions

RJB, YH, and IY collected the data. DeL, RJB, DoL, and YH analyzed the data. RJB and DeL wrote the manuscript. OH markedly contributed to the concept of the manuscript. All authors agreed with the content of the manuscript.

## Conflict of Interest

The authors declare that the research was conducted in the absence of any commercial or financial relationships that could be construed as a potential conflict of interest.
